# An Anatomical Study of the Feasibility of the Pectoralis Minor Transfer for Subscapular Deficiency: An Investigation of the Association Between the Transferred Muscle and the Musculocutaneous and Axillary Nerves

**DOI:** 10.7759/cureus.73031

**Published:** 2024-11-05

**Authors:** Apostolos Gantsos, Dimitrios Giotis, Christos Konstantinidis, Felipe Reinares, Apostolos Polyzos, Odysseas Papazachariadis, Konstantina Moraiti, Emilios Ε Pakos, Nikolaos K Paschos, Philippe Valenti

**Affiliations:** 1 Department of Orthopaedics, General Hospital of Naousa, Naousa, GRC; 2 Department of Orthopaedics, General Hospital of Ioannina "G. Hatzikosta", Ioannina, GRC; 3 Paris Shoulder and Elbow Unit, Institut de la Main, Clinic Jouvene, Paris, FRA; 4 Paris Shoulder and Elbow Unit, Institut de la Main, Clinic Jouvenet, Paris, FRA; 5 Orthopaedics and Biomechanics, Medical School, University of Ioannina, Ioannina, GRC; 6 Department of Orthopedic Surgery, Massachusetts General Hospital, Harvard Medical School, Boston, USA

**Keywords:** axillary nerve, coracoid process, musculocutaneous nerve, pectoralis minor, transfer

## Abstract

Introduction

The purpose of this study was to evaluate the feasibility of transferring the pectoralis minor (PM) in its entirety and assess its relationship with the musculocutaneous nerves (MCN) and axillary nerves (AXN).

Methods

Sixteen fresh transthoracic cadaver specimens were used. After PM transfer, the following measures were obtained: (a) the distance between the coracoid process (CP) and the subscapularis insertion on the lesser tubercle during external, neutral, and internal rotation. (b) The distances between the CP and PM, PM and musculocutaneous nerve, and PM and the axillary nerve. All measurements were performed using a precision caliper by two independent and blind-to-each-other findings observers.

Results

The median distance between the CP and PM muscles was 24 ± 7.7 mm, while the width of the coracoid was 24 ± 5.2 mm. PM-MCN distance was 23.25 ± 21.9 mm, CP-MCN distance was 72.1 ± 32.4 mm, and PM-AXN distance was 4.9 ± 0.7 mm. The distance between the coracoid process and lesser tuberosity varied by rotation: 29.2 ± 5.5 mm in internal rotation, 41.1 ± 8.9 mm in neutral rotation, and 51.1 ± 10.7 mm in external rotation. The distance significantly increased between internal and neutral or external rotation (p <0.05), but no significant difference was observed between neutral and external rotation (p > 0.05).

Conclusion

The distance between the coracoid process and lesser tuberosity increased considerably only between internal and neutral or external rotation positions. Additionally, the importance of identifying the musculocutaneous and axillary nerves and their branches when transferring the pectoralis minor should be highlighted.

## Introduction

Tears of the subscapularis tendon represent a common cause of shoulder dysfunction, with a rate ranging from 27% to 43% [1,2]. The subscapularis tendon structure is critical for balancing the muscular forces of infraspinatus and supraspinatus around the glenohumeral joint, as it is considered the basic rotator cuff musculotendinous unit [1]. Therefore, the insufficiency of the subscapularis tendon unit suggests that it loses its capability of balancing forces onto the humeral head, creating functional disability of the shoulder [3]. Surgical management of these tears has widely been investigated, and favorable results for both open and arthroscopic approaches have been demonstrated [4,5].

Rotator interval manipulations during arthroscopic or open surgery have been shown to be associated with degenerative changes in the anterior rotator cuff tendon units, occurring more commonly in patients with a reduced acromiohumeral distance and impingement [4]. In reparable subscapularis tears associated with irreparable tears of the supraspinatus, restoration of the force couple by partial repair of the anterior and posterior bridles of the cuff re-establishes shoulder balance, but functional restoration is not always associated with strength recovery [6]. Techniques of tendon transfers that involved pectoralis major tendon transfer were elaborated to treat irreparable subscapularis tears. Nevertheless, this technique appears not to be effective in improving shoulder range of motion, such as anterior flexion and external rotation, and the subscapularis tests show its inadequacy [7]. Thus, a technique that involved pectoralis minor (PM) transfer was proposed as an alternative that could combine some of the advantages of the previous technique, but would lack the disadvantages associated with the use of pectoralis major.

Two ways were described for the pectoralis minor tendon transfer. The tendon unit can be transferred beneath the conjoined tendon or rerouted superficially, with the first method considered biomechanically superior [8]. When the transfer is routed beneath the conjoined tendon, this might reduce subcoracoid impingement through soft-tissue interposition, which contributes to pain relief.

These techniques could be applied to isolated subscapularis tears, subscapularis tears combined with anterior supraspinatus tears, or alongside posterior cuff repair for posterosuperior tears. Moreover, they have been used to address subscapularis insufficiency following failed subscapularis repairs after open shoulder stabilization or shoulder arthroplasty. The pectoralis minor tendon was initially used as a graft to treat irreparable subscapularis tears from Wirth and Rockwood, along with the use of the coracoacromial ligament, because of its continuity with the insertion of the pectoralis minor in the coracoid process (CP) [3]. If subcoracobicipital transfer is being considered, the considerable variability in how the musculocutaneous nerve (MCN) approaches penetrates and innervates the coracobrachialis, brachialis, and biceps brachii muscles should always be taken into account, as this could influence the surgical technique.

The objective of the current study is to investigate whether open pectoralis minor transfer is a feasible procedure to restore irreparable lesions of the subscapularis tendon. Additionally, it aims to clarify the relationship between the transferred muscle, the coracoid process, and the musculocutaneous nerve. We hypothesized that a pectoralis minor transfer to address subscapularis tendon deficiency could be conducted effectively beneath the conjoined tendon without impacting the axillary nerves (AXN) or musculocutaneous nerves.

Data from this article was previously presented as a meeting abstract at the 10th Biennial ISAKOS Congress on June 7-11, 2015.

## Materials and methods

Sixteen shoulders (8 left and 8 right) from 12 fresh transthoracic cadaver specimens (6 females and 6 males, mean age 63.4 ± 5.1 years) were used. All cadavers had normal shoulder joints, specifically the critical parts such as the proximal half of the humerus, the clavicle, the coracoid, and the acromion, while the muscles and the ligaments were included intact with no history of previous surgery in all specimens. The study was performed in one institution and was conducted according to previous anatomic studies. The institute obtained all necessary regulatory requirements for handling cadaveric specimens from the institutional review board.

Anatomical dissection

**Figure 1:**
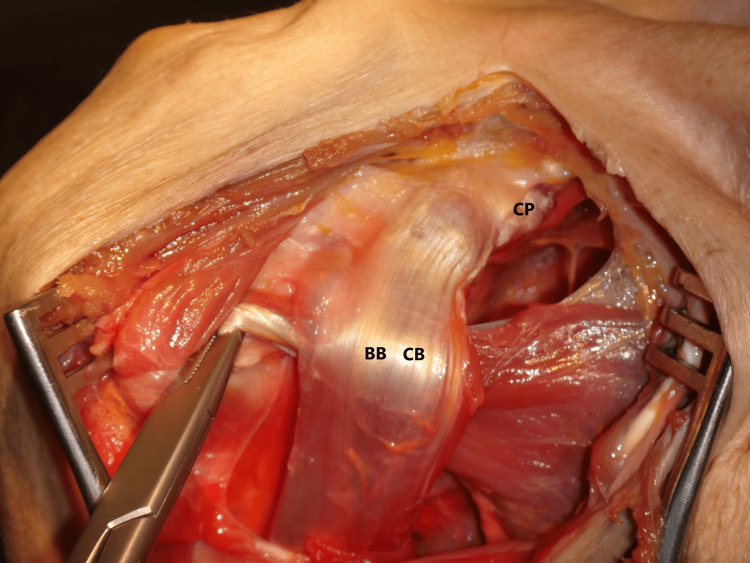
Anatomical cadaver shoulder dissection via deltopectoral approach. The conjoined tendon has been exposed and the inferior border of the coracoid process (CP) has been identified. The pectoralis minor has been detached from the CP, its tendon has been pointed out with forceps and displaced below the conjoint tendon into the lesser tuberosity. BB: biceps brachii, CB: coracobrachialis.

The anatomical dissection was performed by one surgeon, who was the same for every case. Each cadaver was positioned in a beach chair position. A deltopectoral approach was performed, and the skin, along with the subcutaneous tissue, was carefully removed. The aforementioned critical units, such as the subscapularis and conjoined tendons, the coracoid process, the bicipital groove, the long head of the biceps brachii, the pectoralis minor tendon, and the axillary and musculocutaneous nerves with their branches, were identified during careful dissection. Next, the insertion footprint of the subscapularis was identified at the lesser tuberosity. After the surgical exploration and preparation of the coracoid process, the pectoralis minor tendon was detached from the coracoid process. Finally debriding the subscapularis footprint and the space between the coracoid process and humeral head, the pectoralis minor tendon was transferred under the coracoid process (Figure 1). 

Muscle transfer

Sutures were placed at the muscles as guides, and forceps were implemented to pull the PM tendon that normally reached the lesser tuberosity with tension beneath the conjoined tendon. The pectoralis minor was released, with the axillary and musculocutaneous nerves identified and protected. Subsequently, the tendon was secured on the lesser tuberosity. The inferior edge of the coracoid process was recognized and marked on the conjoined tendon. The musculocutaneous nerve and any identifiable proximal muscular branches traced to where they penetrated the conjoined tendon were isolated. Anatomical variations in this area were noticed.

Measurements

**Figure 2:**
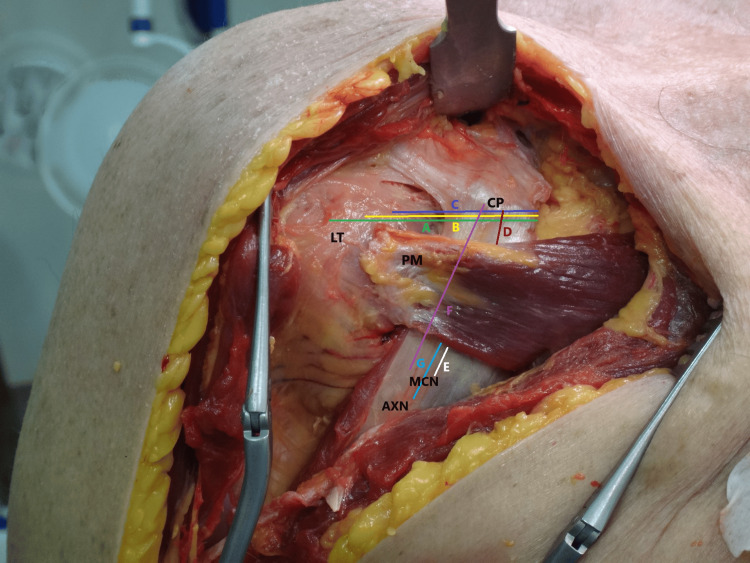
Anatomical specimen with the transfer in place in order to make the examined measurements. The following landmarks were recognized: the inferior border of the coracoid process, the entry point of the most proximal muscular branch of the musculocutaneous nerve in the conjoined tendon, the cranial edge of the pectoralis minor at the medial edge of the conjoined tendon and the caudal edge of the pectoralis minor at the same level. CP: coracoid process, LT: lesser tuberosity, PM: pectoralis minor, MCN: musculocutaneous nerve, AXN: axillary nerve, Distance A (CP-LT) External rotation, Distance B (CP-LT) Neutral rotation, Distance C (CP-LT) Internal rotation, Distance D (CP-PM), Distance E (PM-MCN), Distance F (CP-MCN), Distance G (PM-AXN).

Immediately after dissection and transfer, the shoulder was positioned in zero degrees of abduction, flexion, and rotation. The distances between the coracoid process (insertion point of the PM) and the subscapularis insertion on the lesser tuberosity were measured in three positions: 30° external rotation (distance A), neutral rotation (distance B), and 30° internal rotation (distance C) (Figure 2).

Additionally, the distances between the coracoid process and the middle of the PM tendon (CP-PM, distance D), PM and the upper part of the musculocutaneous nerve (PM-MCN, distance E), coracoid process and musculocutaneous nerve (CP-MCN, distance F), and PM and the upper part of the axillary nerve (PM-AXN, distance G) were also measured. Each measurement was achieved with the use of a manual calliper and was accomplished by two independent observers that were blind to each other's findings.

Statistical analysis

The values obtained were tested by performing comparative analysis with Mann-Whitney tests. The statistical analysis was conducted using SPSS software (SPSS Inc., an IBM Company, Chicago, Illinois). Statistical significance was set at p=0.05. The high ICC (Calculate Intraclass Correlation Coefficient) (ICC=0.90) that was observed indicated a very high level of consistency between the two observers.

## Results

In every specimen, the investigated anatomical landmarks were successfully found, the transfer was performed, and all measurements were recorded. The distance D (PM-coracoid) was 24 ± 7.7 mm, and the width of the coracoid process was 24 ± 5.2 mm. Concerning the musculocutaneous nerve, distance E (PM-MCN) was 23.25 ± 21.9 mm, while distance F (CP-MCN) was 72.1 ± 32.4 mm. Distance E varied greatly, and many distance variations between MCN and the conjoint tendon were observed. More specifically, no significant variations were detected at the pectoralis major, subscapularis, axillary nerve, or the conjoined tendon. However, considerable anatomical variability was found in the musculocutaneous nerve, especially regarding the relation to the conjoined tendon. It was also observed that in 10 specimens (62.5%), at least one proximal muscular branch exited the musculocutaneous nerve before it entered the conjoined tendon. Distance G (PM-AXN) was 4.9 ± 0.7 mm for the axillary nerve. 

The distance between the coracoid process and the lesser tuberosity was 51.1 ± 10.7 mm in external rotation (distance A), 41.1 ± 8.9 mm in neutral rotation (distance B), and 29.2 ± 5.5 mm in internal rotation (distance C) (Table 1). No differences were noticed between males and females.

**Table 1:** Descriptive statistics concerning the recorded measurements. SD: standard deviation, CP: coracoid process, LT: lesser tuberosity, PM: pectoralis minor, MCN: musculocutaneous nerve, AXN: axillary nerve. Measurements: in millimeters.

	Mean	SD	Minimum	Maximum
Distance A (CP-LT) external rotation	51.1	10.7	36	67
Distance B (CP-LT) neutral rotation	41.1	8.9	27	52
Distance C (CP-LT) internal rotation	29.2	5.5	21	40
Distance D (CP-PM)	24	7.7	14	38
Distance E (PM-MCN)	23.25	21.9	8	46
Distance F (CP-MCN)	72.1	32.4	31	110
Distance G (PM-AXN)	4.9	0.7	4	6

Comparative analysis using Mann-Whitney tests revealed a significant increase between internal and neutral or external rotation positions (p < 0.05). However, no statistically significant difference was found between neutral and external rotation positions (p > 0.05).

## Discussion

The subscapularis muscle is the greatest rotator cuff, playing a considerable role in shoulder stability and function. This study tries to demonstrate whether this alternative tendon transfer is feasible and safe as an extra tool in the shoulder surgeon's hands to restore the insufficiency of this great shoulder stabilizer.

Tendon transfers, such as pectoralis major or minor, are procedures that are considered alternatives for subscapularis deficiency, chronic tear, retraction, and fatty degeneration. Pectoralis minor is a shoulder girdle muscle located beneath the pectoralis major, which is attached to the medial part of the coracoid process and originates from the anterior and upper surfaces of the third to fifth ribs, specifically near their cartilages and from the aponeuroses covering the intercostalis. Its innervation comes from the medial pectoral nerve, which pierces also the clavipectoral fascia [9]. Arterial supply originates, in most cases, directly from an axillary artery branch [9]. Additionally, facial reanimation surgery is a field where the PM muscle has been used as a free vascularized, innervated, functional muscle graft for over 20 years [1,10].

Rotator cuff tears are considered the prevalent origin of shoulder morbidity tears. However, the subscapularis tendon has not been acknowledged as the frequent cause [11,12]. Several reports have shown the prevalence of isolated subscapularis tendon tears, especially of the proximal tendinous part, ranging from 2.1% to 10.5% [11,12]. Given the rarity of diagnosis of subscapularis tendon tears, despite its importance as a major muscle of the rotator cuff, the subscapularis has largely been ignored. Since 1991, when the first large clinical series of isolated subscapularis tendon tears was presented, increasing attention has been directed to this clinical problem [13].

There has been a significant debate regarding the feasibility of transferring the pectoralis minor tendon to the lesser tuberosity along its natural path. Since muscle strength is directly reciprocal to muscle mass, this tendon transfer may be compared to the transfer of the split pectoralis major (clavicular and sternal heads). While a complete transfer of the pectoralis major is regarded as too invasive, transferring the pectoralis minor tendon is less invasive but equitably safe and avoids continuing morbidity [14]. Moreover, pectoralis minor transfer is implemented just under the coracoid process and beneath the conjoint tendon, and its unit is less bulky. Furthermore, pectoralis major transfer is performed below the coracoid process, which is a much higher risky area, involving neurovascular bands such as the musculocutaneous nerve [15,16]. Nevertheless, transferring the superior 2 to 3 cm of the tendon, which includes parts of both the anterior and the posterior laminae, may be preferable to the pectoralis minor tendon transfer for several grounds [3]. First, the muscle-tendon unit provides sufficient length due to the greater excursion of the muscle. Second, the cross-sectional area and bulk of the muscle create a more effective checkrein during arm abduction and external rotation. Finally, the larger muscle helps to dampen movement during active motion.

In this study, peculiar variations were detected according to the branches of the musculocutaneous nerve beneath the conjoint tendon, which is similar to the literature. In over 90% of the cadavers, proximal muscular branches exiting the musculocutaneous nerve were found [17,18]. However, the distance between the coracoid process and the MCN branches that were detected implied considerable variability. Our data are similar to those observed by Latarjet reporting an interval between 20 and 120 mm [19], Klepps et al. (44 ± 15 mm, interval 21-90 mm) [18], Ozturk et al. (41.5 ± 11.5 mm, interval 17-72 mm) [20], and Ruiz-Ibán et al. (54.2 ± 33.2 mm, interval 17-156 mm) [21]. All four studies used the most proximal muscular branch of the MCN nerve as a reference for measurements. When the MCN nerve itself is used, the distances are slightly greater: measured at 61 ± 15 mm (interval 35-100 mm) and 62 ± 14 mm (interval 32-104 mm), as reported by Klepps et al. and Ozturk et al., respectively [18,20].

Furthermore, an association between subscapularis tendon avulsion and recurrent shoulder subluxation or dislocation has been occasionally noted [22]. Reporting the findings of the complication management after a failed Bristow procedure, 15 out of 31 requiring a revision operation, it was described that seven of them were notably complicated by severe thinning and fraying of the subscapularis tendon, anterior glenoid erosion, or posterior subluxation [22]. To reinforce this weakened subscapularis, the pectoralis minor was transferred in four patients, while in one patient, the transferred muscle was the pectoralis major. The outcomes in this study were rated as good or excellent in four of the five patients. Despite the fact that the subscapularis was not fully detached in these cases, the promising early results of pectoralis minor transfer led to its use in patients who had a complete loss of the subscapularis muscle [22,23].

Some authors have advocated transferring the pectoralis minor from its coracoid insertion to the greater tuberosity of the humerus to support the anterior structures and balance the external rotators in patients who had suffered an anterior shoulder subluxation or dislocation. Dickson and O'Dell based their approach on phylogenetic studies, which showed that the pectoralis minor tendon inserted on the greater tuberosity of the humerus contributes to the internal rotation of the limb [23]. The superior two-thirds of the subscapularis coalesce into a tendon and attach to the lesser tuberosity, whereas the inferior third remains muscular in its insertion [24]. This characteristic can explain the location of the subscapularis tear in the upper part of the tendon.

This study has the limitation that it is a cadaveric analysis. Therefore, it is challenging to fully assess the clinical relevance of contact between the nerve branches and the muscle. However, the distances recorded in this anatomic report are important in both surgical planning and execution. Avoiding tight transfers is crucial, as they have been associated with temporary musculocutaneous nerve palsy. Thus, surgeons performing a subcoracobicipital pectoralis minor transfer should be aware that this might not be feasible in all cases. Careful dissection is essential, and unique anatomical findings in certain patients may require adjustments to the surgical technique. Undoubtedly, nerve variability should be considered, and time must be taken to identify the AXN and MCN nerves and their branches before completing the transfer.

## Conclusions

Pectoralis minor transfer can be considered an acceptable alternative for subscapularis insufficiency. The distance between the coracoid process and the lesser tuberosity increases considerably from internal to neutral or external rotation but with no significant difference between neutral and external rotation. Additionally, the distance between pectoralis minor and musculocutaneous nerve branches can notably vary. At the same time, it is important to identify the musculocutaneous and axillary nerves and their branches. If a subcoracobicipital transfer is not feasible, a more superficial transfer should be taken into consideration.
